# Setting up a COVID-19 care facility at a prison: An experience from Pakistan

**DOI:** 10.1016/j.amsu.2020.06.043

**Published:** 2020-08-13

**Authors:** Mahmood Ayyaz, Usman Ismat Butt, Muhammad Umar, Wasim Hayat Khan, Muhammad Waris Farooka

**Affiliations:** a Services Institute of Medical Sciences, Lahore, Pakistan; b Surgical Unit 1, Services Hospital, Jail Road, Lahore, Pakistan

## Abstract

Since its emergence from Wuhan at the end of last year, the novel coronavirus has spread to almost every country of the earth posing a significant challenge to healthcare systems everywhere. This article presents a practical model adopted in light of the WHO guidelines which was used by our team to set up the facility and care for the 69 COVID-19 prisoners within the prison itself. In addition to the challenges posed by the COVID-19 the healthcare team also had to overcome the challenge of the unique nature of the setup. The purpose of this article is to describe our response to help providers tasked with caring for prisoners especially in lower socio-economic countries.

COVID-19 was first detected in Wuhan, China in December 2019 [1]. It was declared as a pandemic on March 12, 2020 by the WHO [2]. While the WHO guidelines on “Preparedness, prevention and control of COVID-19 in prisons and other places of detention” were being issued on March 15, 2020 [3] we were coming face to face with a similar situation at Camp Jail (District Jail Lahore) in Lahore in Pakistan.

Camp Jail is an almost 90 year old facility established by the British in the Indian Subcontinent before partition. Located on one of the main roads of Lahore, Ferozpur Road, it is one of the biggest prisons in the province of Punjab. Comprising of 22 barracks it has a sanctioned strength of 1050 prisoners but usually houses more than 3000 inmates [4].

A passenger who planned to return to Italy on March 8, 2020 was intercepted by the Airport Security Force with a significant quantity of contraband at Lahore Airport. He was interned at Camp Jail, Lahore where on March 18, 2020 he began to show flu like symptoms which rang alarm bells. He had been in and out of a couple of barracks which resulted in him in being close proximity to over 500 prisoners. Our hospital being in close proximity to the prison facility was tasked to setup a COVID-19 care facility at Camp Jail, Lahore to deal with a possible outbreak. Our team from the hospital visited the Camp Jail to evaluate the situation and plan further accordingly.

As per the WHO guidelines [3,5] a joint meeting (Picture 1) was held with the Jail authorities. After considering the situation a number of joint decisions were taken and implemented. As a measure to prevent the spread of the disease it was decided to discontinue the induction of further prisoners into the Jail until the situation was resolved. Contact tracing of the confirmed case was done and 527 suspected patients including prisoners and Jail staff were identified. Since they couldn't be released or treated off site, the decision to carry out onsite management was taken. In order to facilitate the care of such a large number of patients it was decided to shift out those prisoners who had no contact with the confirmed or suspected patients. It was decided that the areas thus vacated would be used to quarantine suspected cases and carryout nasopharyngeal swabbing of the suspected cases in line with the local guidelines. The goal was to set up a facility to provide management for asymptomatic and stable patient while our hospital was to serve as referral center if any patient's condition worsened.

In the wake of the extending pandemic the entry point of the Jail had already been restricted with screening via means of thermal temperature guns. All visitors had to wash their hands before entering into the jail and sanitizers had been provided at the entry of each further building within the premises. Visits had been limited. Mobility within the Jail had already been restricted. A detailed log of all movements was already being maintained.

A thorough inspection of the Camp Jail was carried out. (Picture 2) The team visited the Jail Hospital and met with the Jail Hospital doctors who appraised the visiting team of the available facilities at the 66-bedded Camp Jail Hospital which was already completely occupied by routine patients. Barracks were identified to set up the required COVID-19 care facility. At the same time single cell isolation area was identified. As decided more than a 1000 non-contact prisoners were shifted to other holding facilities to enable some space.

With the shifting of the decided prisoners, the setting up of the designated areas was started. 100 beds were shifted from our hospital to facilitate the setting up of an entirely furnished COVID-19 care facility within the Jail. Beds were widely spaced. A team of experts from our hospital visited the Camp Jail to provide donning and doffing training to the jail personnel including duty doctors. They were also educated regarding the basics of management of COVID-19, sample taking techniques and the safe use of personal protection equipment. Nasopharyngeal swab testing was started the same day by a team from the Primary and Secondary Healthcare Department. As the labs were not initially geared to deal with a large number of samples, initial results were slow to roll in.

By 31st March, sampling was in progress by personnel of Primary and Secondary Healthcare Department in collaboration with the jail healthcare professionals supplemented by personnel from our hospital. At the same time 194 single cells were readied to hold the prisoners in isolation till their reports were available. From the common barrack the prisoners would be brought to the testing area for the nasopharyngeal sampling. (Picture 3) After the sample was taken, the prisoners were isolated in single cell till the result of their test were available. Those who tested positive were shifted to the COVID-19 ward while those who tested negative were shifted to another barrack where they were kept till they completed two weeks of quarantine [3].

Keeping in view of the WHO guidelines [3,5] of minimum personal protection for the healthcare providers our hospital provided equipment support to Camp Jail by providing Hazmat Suits, surgical gowns, surgical gloves, respirators, eye protection goggles, caps, hand sanitizers, gloves, blankets and drip sets. Separate area for donning and doffing was completed by 2nd April 2020 (Picture 4) and the task of the disposal of the waste generated was also taken up by our hospital.

After initial negative results of a hundred patients, by 5th April the first positive cases were reported. Those testing positive were shifted from isolation to COVID-19 ward. (Picture 5) As the cases started increasing doctors from our hospital were also deputed at Camp Jail to bolster the hospital medical team and the doctors designated the Primary and Secondary Healthcare Department. Twice a day consultant round was also done. Associate Professor of Infectious Diseases from our hospital and her team also visited the Jail to provide useful feedback for the management of the patients. Repeated sessions of training, personnel support and provision of equipment were provided.

By 9th April all 527 contacts had undergone nasopharyngeal swabbing. There were 59 positive patients who were shifted to the Jail COVID-19 ward. Round the clock health care facilities were provided to the patients by the doctors and consultants deputed from our hospital. Daily physical checkups were done. At the same time the provision of special disposable high nutrition meals thrice a day for these prisoners was done. (Picture 6).

The stress and burden of the disease has been felt even by the best of the healthcare systems. [6]. The World Health Organization recognizes that prisoners are more likely to be affected by the COVID-19. This is because of the restricted movements and close contact the prisoners have with each other. These places thus serve not only to increase the infection among prisoners but can also serve to spread the disease among community when asymptomatic prisoners are released into the community. [7,8]. The situation is even graver in a low-socio-economic country like Pakistan where there is already rampant overcrowding in the prison system along with a poor healthcare system in prisons. Furthermore a large number of prisoners already suffer from co-morbidities like tuberculosis, drug addiction, HIV, hepatitis and diabetes. [9].

As a result of systematic testing a total of 59 positive Covid-19 patients were detected. All of them were managed in the on-site Covid-19 facility. Thankfully all of them remained stable with none requiring any additional support. Despite some concerns [10] by May 2, 2020, all of these 59 had recovered fully without any morbidity or mortality.

Further ten Covid-19 prisoners were shifted to the Covid-19 Facility at this prison and by 8th May all of them had recovered without any incident.

## Conclusion

Managing Covid-19 in an overcrowded, resource-constrained environment is a challenging task. The large number of players involved in the effort requires a lot of co-ordination and persuasion. Education, guidance and leading by example might often be necessary. The team must be prepared to face and fight problems as they arise. Success can be achieved by planning, co-ordination and facing problems as they arise.

Provenance and peer review.

Not commissioned, externally peer reviewed.

### Statement of consent

All staff present in pictures consented to their photo being included and the institution accepts responsibility for this.

Picture 1 meeting between hospital team led and jail superintendent.
picture 2 reviewing the jail facilities.
picture 3 nasopharyngeal swabbing in the jail premesis
picture 4 donning and doffing areas.
picture 5 covid-19 ward set up in jail.
Picture 6 Meals provided to the prisoner patients during their stay at COVID-19 care facility in Jail.

### Time line of key events

Setting up COVID-19 care facility in Camp Jail in Pakistan.

**Table:** 

DATE	EVENT
DEC 2019	COVID OUTBREAK IN CHINA
8^TH^ MARCH 2020	PATIENT ZERO BROUGHT TO CAMP JAIL
2^TH^ MARCH 2020	WHO DECLARED COVID-19 AS PANDEMIC
15^TH^ MARCH 2020	WHO ISSUES GUIDELINES ON COVID-19 MANAGEMENT IN PRISONS
18^TH^ MARCH 2020	PATIENT ZERO DEVELOPS SYMPTOMS AND LATER TESTS POSITIVE
27^TH^ MARCH 2020	EFFORTS TO DEVELOP COVID-19 FACILITY IN JAIL STARTED
31^ST^ MARCH 2020	SYSTEMATIC TESTING OF ALL 529 CASES STARTED
5^TH^ APRIL 2020	1^ST^ POSITIVE CONTACT DETECTED
9^TH^ APRIL 2020	ALL 527 CONTACTS TESTED/59 TEST POSITIVE FOR COVID-19
MAY 2, 2020	ALL 59 PATIENTS RECOVER UNEVENTFULLY IN JAIL FACILITY
